# Stable Hygiene

**Published:** 1895-11

**Authors:** W. R. Claussen

**Affiliations:** Waupaca, Wis.


EDITOR OF THE JOURNAL OF COMPARATIVE MEDICINE, ETC.

In your August issue is published a paper on "Stable Hygiene," by Dr. J. W. Sallade. While there are many good suggestions in that article, I do not agree with the doctor's mode of ventilation. He says on page 481: "A stable should be high and roomy and provided with ventilators in the ceiling, or top ventilation, to carry off the obnoxious gases and effete products." Now let us see what the result of such ventilation would be according to that plan. The carbonic acid is given off in the lungs, while the oxygen of the atmosphere is taken up by the blood. This is true; but the carbonic acid gas given off is more than one-half as heavy again as ordinary air, its sp. gr. being 1.529, consequently it sinks in the atmosphere and occupies the space next to the floor. Now we will suppose the stable is air-tight to the ceiling, but in the ceiling is found a ventilator. How could the carbonic acid be expelled through that ventilator? The consequence of such ventilation would be the poisoning of the cattle and their sure death for want of oxygen, because the carbonic acid expelled from the lungs of the animals would, in a short time, occupy the whole stable.

The top ventilation spoken of is all right; but, in addition to that, there must be some means of egress near the floor for the escape of the poisonous carbon dioxide. This, however, should be arranged in such a way as to cause no direct draught. Of course, the air in stables, as we see them here in the West, can never be very badly contaminated by carbon dioxide for the reason that they are generally frame buildings with doors that are opened several times daily; but I have seen stables in connection with distilleries in the old country wherein the cattle were packed as closely as they could stand. These buildings are brick structures and are (or were in those days) cleaned out once daily, in the morning. After this cleaning the door was shut and the hot barm was pumped into the troughs running in front of the cattle. The only means of escape, in these stables, for the carbonic acid gas was through the gutter leading into the yard, in which the manure was dropped. If this small opening, perhaps one foot square, had been closed, I see no reason why the cattle should not have suffocated.

In my opinion we must, in order to have perfect ventilation, have free circulation of air, and that can never be obtained by top ventilation alone unless a draught, sufficiently strong to carry the carbon dioxide with it, can be obtained. But by having escapes properly arranged at the floor the impure exhalations may be got rid of, while the pure, fresh air can be admitted through ventilators located higher up. If top ventilation alone is desired the ventilator should reach the floor, or nearly so, and some means provided to furnish sufficient draught for the purpose of ventilation. How this could be obtained without some system of heating, with hot and cold air flues, I am unable to say.

An old well, in which choke-damp (carbonic acid) has collected, will give said writer a good illustration of his mode of ventilation. These wells, although open at the top, are dangerous to enter and have caused the death of many a human being.

The Upas Valley of Java is another example. This valley is about three-quarters of a mile in circumference and about thirty feet deep. There is here ample top ventilation, but carbon dioxide covers the bottom to a depth of about eighteen feet and the whole valley-bottom is devoid of vegetation and animal life. If the $CO_2$ could escape respirable air would immediately fill its place.

Yours, with best wishes,

W. R. CLAUSSEN.

WAUPACA, WIS., Sept. 10, 1895.

